# Exploring the Quality of Historic DNA From Pinned Beetles

**DOI:** 10.1002/ece3.74137

**Published:** 2026-08-14

**Authors:** Daniel Lukic, Malte Petersen, Jeel Patel, Rama S. Krovi, Dirk Ahrens, Roland Gerstmeier, Jonas Eberle

**Affiliations:** ^1^ Department Environment & Biodiversity Paris Lodron University Salzburg Austria; ^2^ Research IT Department University Hospital Bonn Bonn Germany; ^3^ University of Bonn Bonn Germany; ^4^ Institute of Systematics and Evolution of Animals Polish Academy of Sciences Krakow Poland; ^5^ Museum Alexander Koenig Leibniz Institute for the Analysis of Biodiversity Change Bonn Germany; ^6^ Zoologische Staatssammlung München München Germany

**Keywords:** contamination, DNA amount, DNA isolation, gappiness, hDNA, museomics

## Abstract

Obtaining fresh DNA samples for biodiversity studies is often challenging. Natural history collections offer an alternative way to obtain data, although the DNA from dry‐preserved specimens of historical collections is typically degraded and potentially contaminated. Here, we tested three standard DNA isolation methods to obtain DNA for genomic sequencing approaches using 20 identical ethanol and dry‐preserved beetle specimens. The best‐performing method was applied to a larger set of fresh and historical specimens representing 74 species of the genus *Trichodes* and 33 species from other genera of Cleridae (Coleoptera) to assess how specimen age and the amount of isolated DNA affect DNA sequence quality measures. Unlike most previous studies, we assessed the quality of isolated DNA through fragment length analysis and whole‐genome sequencing. The here used silica‐column based oligo isolation protocol for short DNA fragments outperformed standard silica‐column based and precipitation‐based isolation methods for historical genomic DNA. Fragment length of isolated DNA slightly declined with sample age in dry‐preserved specimens, whereas the amount of isolated DNA remained unaffected. Higher DNA amounts led to significantly more recovered loci and longer multiple sequence alignments with fewer gaps. Contamination levels were significantly higher in dry‐preserved samples. Our findings demonstrated the suitability of commonly available DNA isolation kits for dry‐preserved museum specimens for next generation sequencing without requiring dedicated cleanroom facilities or hazardous reagents. Compared to sample age, the absolute amount of isolated DNA had higher impact on sequence quality and substantial genomic sequence information could be recovered even from old dry‐preserved specimens. Our results underscore the potential of historic insect specimens as a source of molecular data with easy to apply DNA isolation protocols.

## Graphical abstract

Dry‐preserved museum specimens offer valuable but degraded DNA for genomic studies, making extraction method choice crucial. We found that an oligo‐focused silica‐column protocol yielded higher‐quality short DNA fragments than standard silica or precipitation methods, and that specimen age mainly reduced fragment length and locus recovery, while DNA quantity strongly improved sequence quality. Despite higher contamination in dry‐preserved samples, substantial genomic information can still be retrieved using accessible extraction kits.

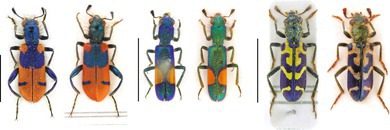

## 1 Introduction

DNA data has become indispensable for biodiversity research: population genetics estimate gene flow, genetic diversity, and population sizes (Rajora 2019), or study the capability of populations to adapt to changing environmental conditions (Allendorf et al. 2022; Hecht et al. 2015), allowing for extinction risk assessments of biodiversity and adequate conservation strategies (Allendorf et al. 2022). Taxonomy and systematics delimit species and refine classifications (Bagley et al. 2015; Grewe et al. 2021), while molecular phylogenetics elucidates evolutionary relationships and processes (Gu 2024).

The acquisition of DNA data has two dimensions: the number of genomic loci and specimens. Both aspects can significantly impact the outcome of a study. Continuous advancements in sequencing technology have further reduced costs, making large‐scale genomic data more easily accessible. However, specimen collection often remains challenging. For instance, phylogenetic studies of widely distributed lineages require researchers to collect specimens across multiple populations or countries, which may present unique challenges due to the rareness of species (Lim et al. 2012), time constraints, bureaucratic hurdles (MacDonald 2025; Watanabe 2015), financial constraints (Britz et al. 2020), or safety concerns. As a result, scientific progress often slows down.

Museomics, that is, the use of DNA from natural history collection specimens, offers a solution to these difficulties. Collections together contain over 1.1 billion samples worldwide (Johnson et al. 2023). Since the approach was first proposed (Gilbert et al. 2007; Miller et al. 2009), museomics applications have been evolving rapidly (Davis and Knapp 2025). Studies across a wide range of vertebrate (Castañeda‐Rico et al. 2020; Huynh et al. 2023; Lyra et al. 2020) and invertebrate taxa (Derkarabetian et al. 2019; Habel et al. 2014; Mayer et al. 2021; Patzold et al. 2020), including beetles (Dietz et al. 2024; Gauthier et al. 2023; Kim and Farrell 2025; Pauli et al. 2024; Toussaint et al. 2021), have demonstrated the immense potential of historic DNA (hDNA) for biodiversity research throughout the majority of metazoan groups. Phylogenetic studies succeeded in including species that were difficult to collect (Kim and Farrell 2025; Lopes et al. 2024). Population genetic studies benefitted from the inclusion of specimens from historic populations, which allowed direct empirical assessments of changes in population structure (Chan et al. 2006; Habel et al. 2014) providing a window to the past and revealing hardly retrievable but very essential information, particularly regarding type specimens. The readily accessible natural history collections could help bridge data gaps and facilitate research on extinct populations or even taxa (Major et al. 2023), thereby enhancing higher quality‐, time‐ and cost‐efficiency of many studies. However, handling collection specimens requires particular care to avoid damage of morphological characters. To minimize structural harm, minimally destructive methods such as using a single leg or thoracic muscles for DNA extraction can help to preserve morphological key traits. This allows for subsequent morphological or further molecular investigation. Ideally, the lab protocol should minimize the need for specialized facilities such as clean rooms or fume hoods (Ferrari et al. 2023), to ensure broad applicability for researchers, including those in smaller laboratories or museums, and taking advantage of the increasing affordability of next‐generation sequencing technologies.

Despite many successful applications of museomics, samples frequently fail to yield usable data, as hDNA extracted from dry‐preserved museum specimens is often highly degraded and thus not in ideal condition for DNA‐based analysis (Raxworthy and Smith 2021). Therefore, researchers continue refining extraction and isolation methods to increase DNA yield (e.g., Holmquist et al. 2025; Lopes et al. 2024; Marinček et al. 2022). The attempts of minimizing destructive approaches for beetle specimens date back almost two decades (Gilbert et al. 2007), motivated by the need to preserve highly valuable museum material and retain most of the valuable external morphological characters. Due to the strongly sclerotized exoskeleton in beetles, dry‐preserved individuals only slowly desiccate during the preservation process after pinning and consequently DNA typically rapidly degrades into small fragments (Mandrioli et al. 2006). Often, resulting fragments are on average less than 100 base pairs long (61–109 bp after 9–138 years; Heintzman et al. 2014). However, a similar DNA degradation has also been observed in less strongly sclerotized bumblebees (Mullin et al. 2023) and butterflies (Zimmermann et al. 2008). The ends of DNA fragments may lose nucleotides through depurination and deamination (Mitchell et al. 2005) and are prone to develop nicks and double‐strand breaks. In previous studies, dry‐preserved beetles exhibited notable variation in hDNA concentration and fragment length (Pauli et al. 2024; Toussaint et al. 2021), and this variability often appeared to be unpredictable. In addition, during the degradation process, the growth of fungi and bacteria within slowly drying specimens can result in significant contamination. However, patterns of DNA deterioration are inconsistent across studies, and contamination levels are insufficiently investigated.

Ultimately, low DNA quality results in difficulties to assemble the data or to map at least portions of the targeted genomic loci to reference sequences, and this leads to a considerable amount of missing data, that is, gappy DNA sequence alignments. The latter are known to cause difficulties in downstream analyses (Darriba et al. 2016; Hutter and Duellman 2023; Sayyari et al. 2017; Simmons 2012). However, the extent to which DNA quantity and sample age affect sequence quality in terms of fragment length, the length and number of recovered loci, and alignment completeness remains largely unknown. A slightly negative correlation of sample age and DNA yield was shown in some studies (Bernstein and Ruane 2022; Hawkins et al. 2022; Roycroft et al. 2022) but could not be confirmed in others (Nunes et al. 2022; Pacheco et al. 2022; Pavlek et al. 2022).

The present study addresses this knowledge gap by providing a quantitative assessment linking sample age and DNA yield to genomic sequence quality of a taxon‐specific set of loci in dry‐preserved historical specimens, to assess which samples are most suitable for downstream analyses. We compared the performance of three commercial DNA isolation kits that are commonly used due to their uncomplicated application and their inferior toxicity compared to, for example, phenol‐chloroform extractions (Gilbert et al. 2007). For enabling direct comparison, each isolation kit was first applied to the same historic, dry‐preserved beetle specimen for which we used minimally destructive extraction methods that preserved its external morphology. DNA degradation and sequencing success were then examined across additional dry‐ and ethanol‐preserved specimens ranging in age from 2 to 118 years. Specifically, we evaluated the amount of DNA yielded, fragment length, and sequencing success in terms of percentage of recovered loci, gappiness (as proxy for alignment completeness) and the total number of recovered nucleotides. Finally, we estimated contamination levels by quantifying the proportion of non‐insect DNA in the extracts.

## 2 Materials and Methods

### 2.1 Specimen Sampling

For the present study, we investigated 131 beetle specimens of the superfamily Cleroidea (82 dry‐ and 49 ethanol‐preserved; Table S1), including 74 species of *Trichodes* and 33 species from other clerid genera (1–5 specimens per species). Dry‐preserved specimens ranged in age from 2 to 118 years, whereas ethanol‐preserved specimens were collected 2–17 years ago. A total of 111 specimens were sent for whole‐genome sequencing. All dry‐preserved and most ethanol‐preserved samples originated from the collection of Roland Gerstmeier (CRG) (Munich, Germany), originally gathered by 66 different collectors. Vouchers are stored in CRG and the beetle collection of the department Environment & Biodiversity at the University of Salzburg (Austria).

### 2.2 DNA Isolation Comparison

**FIGURE 1:**
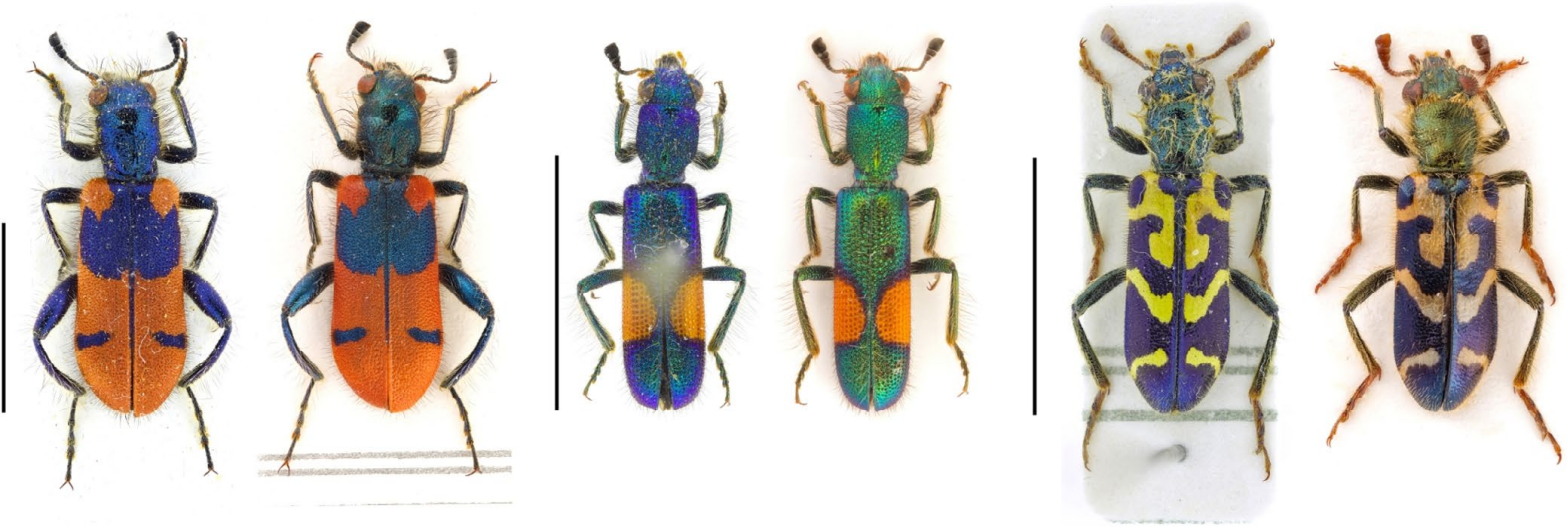
Representative image pairs of dry‐preserved specimens of three species (from left to right: *Trichodes ephippiger*, *T. penicillatus* , and *T. ornatus* ), before (left) and after (right) DNA extractions. Scale bars = 0.5 cm.

Initially, we conducted a comparative evaluation of three DNA isolation methods using the Qiagen DNeasy Blood & Tissue kit (hereafter referred to as the “standard silica‐column”), the Monarch PCR & DNA cleanup kit (“oligo silica‐column”), and the Idylle DNAbsolute kit (“precipitation”) on a total of 20 specimens (13 dry‐ and 7 ethanol‐preserved). The selected methods included a commonly used standard silica column approach, a silica column approach developed for short DNA fragments (e.g., oligonucleotides or PCR amplicons), and a precipitation‐based method to also represent a non‐column‐based approach. The samples were externally cleaned by submersion in distilled water for 20 s, followed by rinsing with 95% ethanol. Stronger decontamination treatments, such as bleach, were deliberately avoided. Although mild, carefully calibrated pre‐lysis bleach rinses can reduce surface contamination without apparent adverse effects on endogenous DNA in insect museum vouchers (Brown et al. 2025) and in dietary and microbiome studies across multiple arthropod taxa (Greenstone et al. 2012; Huszarik et al. 2023; Linville and Wells 2002), the optimal bleach treatment appears to be precarious, as bleach can penetrate tick tissues and impact the internal microbiota (Fernández‐Ruiz et al. 2023). As this study did not aim to distinguish external from internal microbial DNA, a simple water and ethanol rinse was considered sufficient for surface decontamination. Remaining bacterial sequences could be readily identified and removed during downstream bioinformatic analyses. To further reduce potential contamination from gut contents, the abdomen of each specimen was removed prior to lysis. The rest of the body was submerged in lysis buffer to extract DNA from the thoracal flight muscle tissue. Lysis was conducted with 540 μL ATL buffer and 60 μL Proteinase K (> 600 mAU/mL; Qiagen). To ensure that all three methods were directly comparable, we used the same lysis reagents for every protocol, allowing us to standardize the lysis process and to use the same specimen across all methods. This approach ensured that any observed differences reflected variations in DNA isolation rather than differences in lysis (i.e., DNA extraction). We tripled the usual 180 μL of the standard protocol, since the lysate was split into three equal aliquots for the subsequent individual isolation protocols. The concentration of the isolates was finally measured using a Qubit fluorometer with 2 μL of sample input, applying the standard 1X assay dilution of the High Sensitivity (HS) dsDNA kit. DNA fragment lengths were measured using an Agilent Fragment Analyzer 5200 with the DNF‐488 HS Genomic DNA Kit, following the standard protocol and using 2 μL of DNA isolate as input. The most abundant fragment length was determined from the electropherograms. To assess differences in the DNA amount between the three isolation methods, a Kruskal‐Wallis test was used, with Bonferroni‐adjusted post hoc tests for pairwise group comparisons. The voucher specimens were then mounted on a card or pinned for storage in CRG. The outer morphology of the beetles was not altered by the extraction procedure (Figure 1).

### 2.3 DNA Quality Measures and Sequencing

Based on the results of the DNA isolation comparison, the Monarch PCR & DNA cleanup kit (“oligo silica‐column”) was used to extract an additional 111 samples (68 dry‐preserved and 43 ethanol‐preserved), following the oligonucleotide cleanup protocol (Patzold et al. 2020). Lysis was done using Qiagen reagents (180 μL ATL buffer and 20 μL proteinase K). The thorax of most specimens was fully submerged in the lysis solution. For four large specimens, the pronotum was detached from the rest of the body so that both parts could be immersed. The volumes of the extraction protocol were adjusted to 400 μL DNA Cleanup binding buffer and 900 μL molecular grade ethanol. DNA was eluted from the column using 100 μL molecular grade water in two steps (50 μL each). The final DNA concentration was measured as previously described. Fragment lengths were measured for 52 dry‐preserved and 9 ethanol‐preserved specimens, following the protocol described above.

Genomic DNA‐extracts of the 111 samples were sent to Novogene (German Sequencing Center, Munich) for paired‐end whole‐genome sequencing on an Illumina NovaSeq X Plus Series. Due to reduced DNA yield from splitting the lysate into three aliquots for method comparison, the 20 samples from the DNA isolation comparison were not submitted for sequencing. To include the maximum number of specimens and to assess the potential of samples with very low amounts of DNA for molecular analysis, we submitted DNA quantities ranging from 7 to 3869 ng, despite Novogene's recommended minimum of 30 ng. We estimated a coverage of approximately 24× at 24 Gb per sample. The genome size of 10 pilot specimens was therefore estimated with Genomescope beforehand (v. 2.0; Ranallo‐Benavidez et al. 2020; Vurture et al. 2017) (Table S2). A set of orthologous groups (OGs) was generated based on additional mRNA sequencing of nine clerid species (*Trichodes affinis, T. quadriguttatus, T. syriacus, T* *. nobilis* , *T. viridiaureus, T. dilatipennis, T. apiarius, T. alvearius*, and *Korynetes caeruleus*), together with the published genome of *Tillus elongatus* (NCBI accession number GCA_964006375.1) (Booth and Crowley 2025) that was annotated with Augustus (v. 3.4; Stanke et al. 2008). mRNA sequencing results were assembled with Trinity (v. 2.15.1; Grabherr et al. 2011) and filtered for a minimum percentage of 20 for the dominant isoform. The resulting assemblies were then translated into amino acid sequences using TransDecoder (v. 5.7.1; Haas; https://github.com/TransDecoder/TransDecoder, accessed 3 July 2024). Ortholog‐groups (OGs) were predicted using Proteinortho (v. 6.3.2; Klemm et al. 2023; Lechner et al. 2011), retaining single‐copy OGs present in at least 50% of the nine species. Raw reads were assembled and extracted based on the OGs using the CAPTUS‐pipeline (v. 1.4.8; Ortiz et al. 2024). Potential contaminant reads were not explicitly removed before assembly, since reference‐guided contig‐extraction excluded them prior to downstream analyses. Based on the resulting alignments, we calculated the mean of the proportion of gaps per locus for each specimen (gappiness), the mean length of recovered bp per reference OG (number of bp) and the percentage of recovered loci from this set (recovered loci).

### 2.4 Effects of DNA Amount and Sample Age on DNA Sequence Quality

Gappiness, number of base pairs, percentage of recovered loci and percentage of target DNA (insect reads were used as a proxy, see below) were evaluated in relation to DNA yield. Sample age was tested for its effect on DNA yield (DNA amount), most abundant fragment length (fragment length), gappiness, number of base pairs (number of bp), percentage of recovered loci (recovered loci) and percentage of target DNA reads. Dry‐ and ethanol‐preserved specimens were analyzed independently in ordinary least squares models for each quality metric in R (v. 4.3.0; R Core Team 2023). Model fits were evaluated via diagnostic plots generated with the performance package (v. 0.12.3; Lüdecke et al. 2021), and parameter estimates were obtained using the parameters package (v. 0.22.2; Lüdecke et al. 2020).

### 2.5 Contamination Estimates

As the majority of our samples were dry‐preserved, a substantial proportion of microbial DNA (e.g., bacteria, fungi, and viruses) was expected, whereas we had no specific expectations for the ethanol‐preserved samples. Levels of contamination were assessed by matching adapter‐trimmed Illumina reads against a database for metagenomic classification (Martí et al. 2024) using Centrifuge (v. 1.0.4.; Kim et al. 2016). Before the analysis the adapters have been trimmed and only reads with a minimum length of 30 bp were kept. Then deduplication was conducted with fastp (v. 1.3.3.; Chen 2025), removing exact duplicates, overrepresented sequences, and low complexity reads that are likely PCR/sequencing artifacts. Thirty‐six dry‐preserved and 18 ethanol‐preserved samples were randomly selected from the dataset to assess taxonomic composition across varying sequence quality levels. Four of the samples have been additionally analyzed without deduplication: three dry‐preserved samples representing high, medium and low quality, as determined by the number of recovered reference loci, were selected; one high‐quality ethanol‐preserved sample was analyzed as a reference. Given that beetles, and *Trichodes* in particular, were underrepresented in the reference database, we inferred target DNA indirectly by using matches to insect taxa as a proxy. Assuming that numbers of raw reads are largely proportional to numbers of DNA fragments in the extracts, we expected a rough quantification of contamination levels.

## 3 Results

### 3.1 DNA Isolation Comparison

**FIGURE 2:**
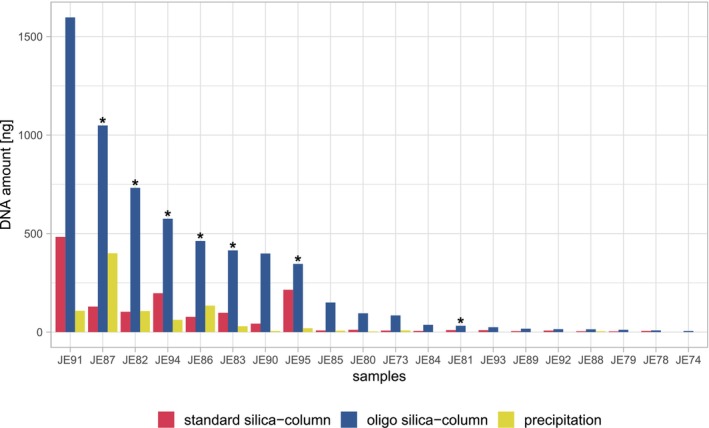
Comparison of DNA yields (ng) from three different DNA isolation kits. Each of the 20 samples was split into three equal aliquots after lysis of entire specimens except abdomen to continue with distinct DNA isolation protocols. Sample IDs correspond to the ones in Table S1. Ethanol preserved samples are marked with an asterisk.

The comparison of DNA yields from the three extraction methods (Kruskal‐Wallis rank sum test: *H* = 14.95, *p* < 0.001; Figure 2) revealed that the oligo silica‐column method resulted in the highest yield of extracted DNA in both dry‐ and ethanol‐preserved samples: it yielded significantly more DNA (median = 89.9 ng, mean = 303.0 ng) than standard silica‐column isolation (median = 9.86 ng, mean = 71.2 ng) (*p* < 0.05) and precipitation isolation (median = 4.67 ng, mean = 44.4 ng) (*p* < 0.01). There was no significant difference between the DNA yield of the standard silica‐column and precipitation‐extracted samples (*p* = 0.19). The DNA yield of the oligo silica‐column samples was 6.82 times higher than that of the precipitation‐extracted samples and 4.26 times higher than that of the standard silica‐column samples. The average DNA yield of samples isolated using the oligo silica‐column was 81.79 ng for dry‐preserved samples and 515.89 ng for ethanol‐preserved samples.

### 3.2 Effect of DNA Amount on DNA Sequence Quality

**FIGURE 3:**
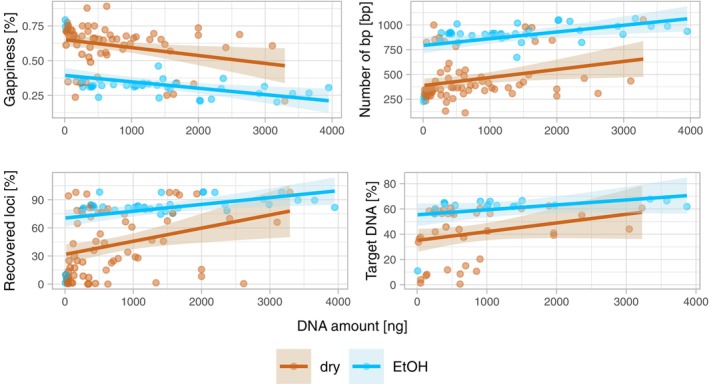
Relationship between the amount of yielded DNA and mean gappiness, mean number of recovered base pairs per locus, percentage of recovered loci and percentage of target DNA proxy (insect reads) for dry‐ and ethanol‐preserved samples. Significant relationships are illustrated by regression lines with standard error estimates.

Based on mRNA sequences and the reference genome, we identified a total of 2897 OGs that served as a reference. Of the 111 samples submitted for sequencing, 109 were successfully processed (98.2%). Reference‐guided extraction and subsequent alignment of sequences from the best‐performing sample (JE157; ethanol‐preserved; see Table S1) against the reference OGs resulted in a total of 3,823,848 base pairs across 2849 loci. Gappiness of alignments significantly decreased with increasing amount of extracted DNA for both dry‐ and ethanol‐preserved samples (Table 1; Figure 3), although a large proportion of the variation in gappiness was not explained by DNA amount ($R_{\mathrm{dry}}^{2}$ = 0.069, $R_{\text{ethanol}}^{2}$ = 0.205). The number of recovered base pairs ($R_{\mathrm{dry}}^{2}$ = 0.064, $R_{\text{ethanol}}^{2}$ = 0.125) and percentage of recovered loci ($R_{\mathrm{dry}}^{2}$ = 0.087, $R_{\text{ethanol}}^{2}$ = 0.123) significantly increased with larger amounts of DNA available for genome sequencing, especially in dry‐preserved specimens (Table 1; Figure 3). Relative abundance of endogenous DNA increased with DNA amount in both preservation types, but the relationship was strongly significant in ethanol‐preserved specimens, whereas it was marginally non‐significant in dry‐preserved specimens ($R_{\mathrm{dry}}^{2}$ = 0.080, $R_{\text{ethanol}}^{2}$ = 0.726). Untrimmed alignments are available as https://zenodo.org/records/17989238.

**TABLE 1:** Relationships of mean gappiness, mean number of recovered base pairs per locus, percentage of recovered loci, and percentage of target DNA proxy (insect reads) with DNA amount.

Pres.	Model	Adj. *R* ^2^	Effect	Effect interpretation	df	*p*
Dry	Gappiness ~ DNA amount	0.069	−5.68 × 10^−5^	Decrease in gappiness [%] per ng DNA	66	< 0.05*
Dry	log(number of bp) ~ DNA amount	0.064	0.016	Percentage change in no. bp per ng DNA	66	< 0.05*
Dry	log(recovered loci) ~ DNA amount	0.087	1.412	Percentage change in no. recovered loci per ng DNA	66	< 0.01*
Dry	Percentage target DNA ~ log(DNA amount)	0.080	0.374	Percentage [unit] increase of target DNA per 1% DNA amount	34	0.053
Ethanol	log(gappiness) ~ DNA amount	0.205	−0.012	Percentage change in gappiness [%] per ng DNA	39	< 0.01*
Ethanol	Number of bp ~ DNA amount	0.125	0.028	Increase in no. bp per ng DNA	37	< 0.05*
Ethanol	log(recovered loci) ~ DNA amount	0.123	0.003	Percentage change in no. recovered loci per ng DNA	37	< 0.05*
Ethanol	Percentage target DNA ~ log(DNA amount)	0.726	0.450	Percentage [unit] increase of target DNA per 1% DNA amount	16	< 0.001*

*Note:* Linear model definitions (in the form response ~ predictor) and metrics are given. Effect sizes are presented as additive changes for linear models, as percentage changes for log‐transformed response variables, computed as $\left(e^{\beta }-1\right)\times 100$, and as additive changes per percentage predictor increase for models with log‐transformed predictors. Significant *p*‐values (*p* < 0.05) are shown in bold and marked with an asterisk.

### 3.3 Effect of Sample Age on DNA Sequence Quality

**FIGURE 4:**
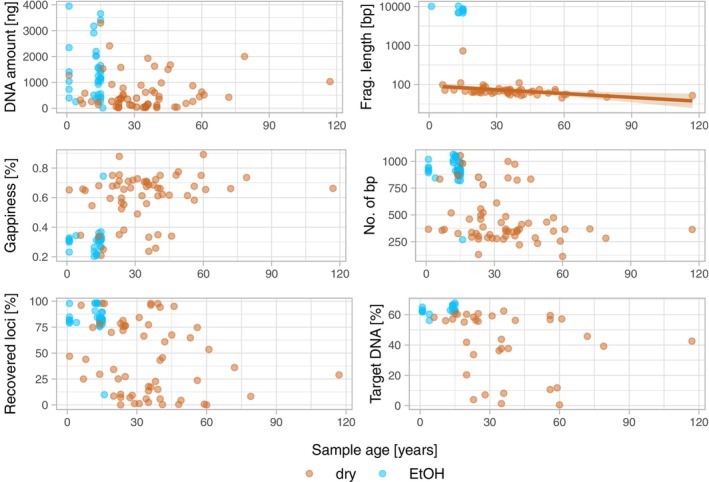
Relationship of sample age and DNA amount, most abundant fragment length, mean gappiness, mean number of recovered bp per locus, percentage of recovered loci and percentage of target DNA proxy (insect reads) for dry‐ and ethanol‐preserved samples. Significant relationships are illustrated by regression lines with standard error estimates.

An interesting result was that sample age did not noticeably influence the amount of DNA recovered. DNA amount, gappiness, recovered loci and percentage of target DNA proxy (insect reads) varied considerably across the sample age range, suggesting that other factors must have had a greater influence (Table 2, Figure 4). We found a small significant negative effect of sample age on fragment length in dry‐preserved samples (Table 2). The mean fragment length of dry samples was 78 bp and decreased by 0.51% per year in samples in the age range of 2–118 years. In dry samples, gappiness increased and the number of recovered loci decreased with sample age, but these changes were not statistically significant (Table 2; Figure 4). In general, although with exceptions, ethanol‐preserved samples performed considerably better than dry‐preserved samples (Table S3).

**TABLE 2:** Relationships of DNA amount, most abundant fragment length, mean gappiness, mean number of recovered base pairs per locus, percentage of recovered loci, and percentage of target DNA proxy (insect reads) with sample age.

Preservation	Model	Adj. *R* ^2^	Effect	Effect interpretation	df	*p*
Dry	log(DNA amount) ~ age	0	0.585	Percentage change in DNA amount per year	57	0.52
Dry	log(fragment length) ~ age	0.261	−0.506	Percentage change in most abundant fragment length per year	49	< 0.001*
Dry	log(gappiness) ~ age	0.036	0.401	Percentage change in gappiness [%] per year	57	0.08
Dry	log(number of bp) ~ age	0.040	−0.576	Percentage change in no. bp per year	57	0.07
Dry	log(recovered loci) ~ age	0.02	−1.758	Percentage change in no. recovered loci per year	57	0.15
Dry	Percentage target DNA ~ age	0.015	0.176	Increase in Percentage [unit] of target DNA per year	29	0.24
Ethanol	log(DNA amount) ~ age	0	−3.354	Percentage change in DNA amount per year	33	0.34
Ethanol	log(fragment length) ~ age	0.152	−1.751	Percentage change in most abundant fragment length per year	7	0.16
Ethanol	Gappiness ~ age	0	5.84 × 10^−4^	Increase in gappiness [%] per year	33	0.69
Ethanol	Number of bp ~ age	0	−0.700	Decrease in no. bp per year	33	0.73
Ethanol	Recovered loci ~ age	0	−0.365	Decrease in no. recovered loci per year	34	0.44
Ethanol	Percentage target DNA ~ age	0.180	−0.217	Decrease in percentage [unit] of target DNA per year	15	0.05

*Note:* Linear model definitions and metrics are given. Effect sizes are presented as additive changes for linear models, as percentage changes for log‐transformed response variables, computed as $\left(e^{\beta }-1\right)\times 100$, and as additive changes per percentage predictor increase for models with log‐transformed predictors. Significant *p*‐values (*p* < 0.05) are shown in bold and marked with an asterisk.

### 3.4 Contamination Estimates

**FIGURE 5:**
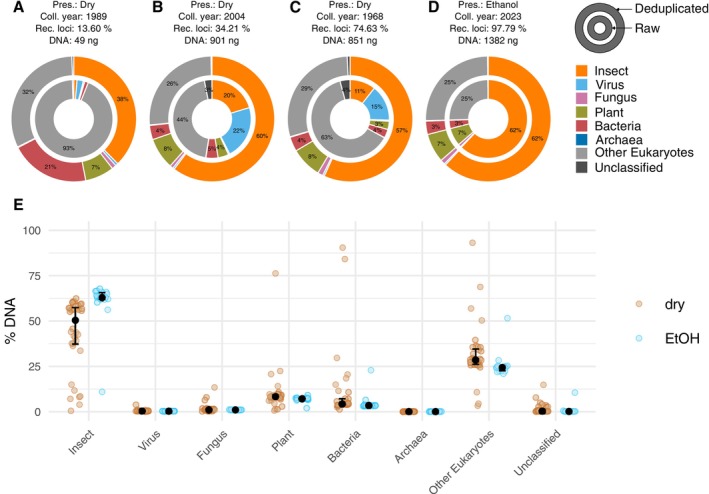
(A–D) Double‐donut visualizations of metagenomic profiles from three dry‐mounted samples (A–C; JE115, JE191, JE217) and one fresh ethanol‐preserved sample (D; JE229), showing the relative proportions of taxonomic groups, where insect reads served as a proxy for the target genus *Trichodes*. The inner ring represents raw data, whereas the outer ring represents data after duplicate removal. Only taxa comprising more than 3% of total reads are labeled. Pres. = preservation method; Coll. year = year of sample collection; Rec. loci = percentage of recovered reference loci; DNA = DNA amount. (E) Distribution of DNA percentages among taxonomic groups in dry‐preserved (*n* = 36) and ethanol‐preserved (*n* = 18) samples.

Eighteen ethanol‐ and 36 dry‐preserved samples were tested for contamination. The dry‐preserved samples, showed strong variation in the proportion of contaminant reads, ranging from 37.39% to 99.26% (Figure 5). In most samples, the largest proportion of reads not assigned to insects was classified as “other eukaryotes”, followed by “plants” and “bacteria”. Most reads that matched these contaminant categories were associated to overrepresented model organisms in reference databases (e.g., *Homo sapiens* , *Triticum aestivum* , *Danio rerio, Bos taurus*). The most abundant taxon across all samples was *Enterobacter cloacae* , a gut‐associated bacterium widely distributed among animals and reported from several insect species, where members of this species complex may contribute to nutrient provision/degradation and to pathogen protection (Stathopoulou et al. 2021). Samples with low initial DNA amount, that is, samples that needed strong amplification during sequencing library preparation suffered from high read proportions of PCR duplicates (Figure 5). Ethanol‐preserved samples contained more target (insect) DNA than dry‐preserved samples (Wilcoxon rank‐sum exact test, *W* = 52, *p* < 0.001). This pattern was consistent across all tested contaminant groups except fungi and archaea (*p* = 0.06 and = 0.10, respectively). The majority of contaminant groups only represented a small proportion of total reads (Figure 5). Variability in the relative abundance of sequence reads assigned to taxonomic categories across ethanol samples was lower compared to dry‐preserved samples, however sample sizes differed between treatments (*n* = 18 for ethanol and *n* = 36 for dry). We could not find evidence for a dependence of the percentage of target DNA sequenced per specimen on sample age in dry‐preserved specimens (Figure 4, Table 2). Higher amounts of DNA, on the other hand, resulted in significantly higher percentages of endogenous DNA (Figure 3, Table 1) in both ethanol and dry preserved samples.

## 4 Discussion

Our quantitative investigation of historical DNA from beetle specimens provided support in favor of using oligo isolation protocols for DNA extraction from dry preserved insects. Although differences between the isolation methods regarding sequence quality in terms of fragment length and sequence completeness were not explicitly tested, we found that samples with higher initial DNA yield resulted in better quality of final data (multiple sequence alignments). Although isolation of short DNA fragments is normally not preferred for fresh and well‐preserved specimens, our results justify this approach for historical samples with degraded DNA.

It has been previously recommended for pinned Lepidoptera to adopt PCR‐amplicon purification protocols for hDNA samples (Djernæs et al. 2026; Patzold et al. 2020), given their capacity to recover short DNA fragments that could otherwise be lost during standard DNA isolation procedures (Marinček et al. 2022). We confirmed this finding by investigating DNA yield from pinned Coleoptera of the same specimens using different DNA isolation kits. Beetle DNA typically undergoes greater degradation during drying in the mounting process because of its stronger body sclerotization (see below). Notably, in our study, oligo silica‐columns recovered both very short and long fragments. Higher amounts of isolated DNA had a significant positive influence on several sequence quality metrics (Figure 3), reducing gappiness, resulting in longer sequences per locus and increasing the percentage of recovered loci and target DNA. Our results once more suggested that maximizing DNA yield by using oligo‐isolation protocols may be advantageous when working with dry‐preserved museum samples. Although we only tested the Monarch kit, PCR‐ and oligo‐cleanup sets from other manufacturers may likewise perform well in recovering large amounts of small DNA fragments.

Our findings also underscore and reconfirm the inherent unpredictability in DNA quality obtained from dry‐preserved museum specimens, due to unknown differences in preservation history, ethanol concentration, other preservation chemicals (Stočes et al. 2025), as well as historical storage and handling conditions. This was reflected in considerable variation in DNA yield among our samples (Figure 4), a trend also reported in previous studies (Djernæs et al. 2026; Pauli et al. 2024; Toussaint et al. 2021) although a more consistent decline in DNA yield was reported from moths (McGaughran 2020). Time‐related DNA degradation could be characterized from our results as follows: After rapid initial fragmentation of genomic DNA in dry‐preserved beetles (shortly after their collection and dry‐preservation), deterioration slowed down once fragment lengths reached approximately 80 bp, with a subsequent mean fragmentation rate of approximately 0.5% per year (Table 1). These results were in line with previous studies (Mandrioli et al. 2006) on historic samples of Coleoptera (Heintzman et al. 2014; Pauli et al. 2024; Thomsen et al. 2009), Hymenoptera (Mullin et al. 2023) and Diptera (Tin et al. 2014). A detrimental trend of age on sequence gappiness and the percentage of recovered reference loci was observable, but not statistically significant. Anyhow, it was promising that even very old specimens, for example, *Trichodes rectilinea* (JE222), collected in the year 1907, yielded large amounts of DNA (1008 ng).

Other factors besides the amount of DNA and sample age that have a strong impact on quality measures certainly include the method to preserve the sample (Dean and Ballard 2001), the duration of the drying process, historical and current storage conditions, and potential post‐mortem re‐softening, which is often practised for genitalia extraction or re‐mounting. Longer periods in moist or even wet conditions foster rotting and lead to DNA degradation in insects (Dessauer et al. 1990; Fowler et al. 2024), especially when combined with warm, fluctuating temperatures and sunlight (Mende and Hundsdoerfer 2013). This may explain why the most abundant fragment lengths in DNA extracts from dry‐preserved butterflies were above 100 bp (Patzold et al. 2020), while it was below 100 bp (mean: 78 bp) in our and other studies on beetles (61–109 bp after 9–138 years; Heintzman et al. 2014). The exoskeleton of many beetles is generally thicker than that of butterflies, potentially slowing the drying process, which may, in turn, increase DNA degradation (Lindahl 1993; Seutin et al. 1991). However, other studies have shown that even rapid desiccation using silica gel can result in substantial DNA fragmentation in freshly collected moths (Mandrioli et al. 2006). Certain preservation methods, such as acidic and formalin‐based fixatives like Scheerpeltz solution or Renner solution (Ruppert et al. 2023), are used to maintain specimen softness for dissection, which can be highly detrimental to DNA integrity. Exposure to acidic conditions promotes nucleic acid depolymerization within tissues, significantly compromising DNA quality (Gimenez et al. 1977). The here investigated samples were collected by 66 different individuals, who likely varied in their sampling and preservation practices. As information on these treatments, as in our case, is typically undocumented, assessing their impact on the DNA quality of historical samples was impossible.

The process of decomposition is driven by microbiota (Harrison et al. 2020), which in dry‐preserved insects likely originate from the endobionts of the insects themselves. We therefore expected higher concentration levels of bacteria and fungi DNA in more degraded samples. Contamination analyses indicated that, although high‐quality ethanol‐preserved samples also contained notable amounts of contaminant DNA, contamination levels were significantly higher in dry‐preserved specimens (Figure 5e). This difference suggests substantial growth of microorganisms during the decay process of pinned and thus suboptimally preserved specimens. Notably, the variation in the amount of contamination was very high in dry‐preserved specimens, once more highlighting the unpredictability of the quality of dry collection samples due to differences in storage and handling conditions (Stočes et al. 2025). Although high amounts of bacterial DNA were expected due to the decay process but also due to gut microbiome, the discovered high percentages of plant and other eukaryotes require further consideration. Most species of Cleridae prey on other insects that they hunt on plants or carcasses. Some species, in particular of the genus *Trichodes*, also feed on pollen. The measured contaminations might therefore actually stem from gut contents and remaining particles on the beetles' surface. As the specimens were rinsed, abdomens were dissected prior to DNA extraction, and because contaminations were significantly higher in historical specimens, methodological artifacts were more likely to be the source of plant and other eukaryote matches. Identifying the actual origin of a sequencing read is not trivial. All reads must be matched against existing DNA databases. Unfortunately, bias toward overrepresented reference taxa is a recurrent failure of commonly used taxonomic assignment procedures. When the true target organism is absent or sparsely represented in the databases, reads are often forced onto the nearest well‐sampled taxon rather than left unresolved, as shown in various studies (Koski and Golding 2001; Marcelino et al. 2020; Ross et al. 2008), which can lead to difficulties in the interpretation of contamination analysis. In addition, conserved genes are not unlikely to match sequences from even distantly related species, in particular when those sequences are present in high quality, continuous stretches and multiple isoforms in the databases. Even reads of endogenous DNA could therefore match to e.g., plants and other eukaryotes.

A further major source of low final sequence quality is to find during the PCR‐based amplification of genomic DNA during sequencing library preparation. Samples with low amounts of DNA need more amplification cycles, which often results in many PCR‐duplicates and therefore low sequencing library complexity (e.g., Ávila‐Arcos et al. 2011; McGaughran 2020) (Figure 5A–D). An apparent threshold in initial DNA quantity was observed, above which the percentage of sequenced endogenous DNA stabilized in dry‐preserved samples (Figure 3). Future studies should further investigate this issue. High amounts of PCR duplicates but also increased levels of contaminants inflate the required sequencing effort. These are problems that might be overcome with targeted sequencing approaches, however, choosing between expensive probe synthesis and deeper sequencing is a trade‐off that might be decided based on the size of the target species' genome.

Although sample drop‐out rates of specimens with very poor DNA quality are often substantial (Andersen and Mills 2012; Prosser et al. 2016), 109 out of 111 samples (98%) were successfully sequenced in our study. In the era of high throughput sequencing and further dropping sequencing costs, it seems more feasible than ever to sequence specimens from historical collections. The data potential inherent in museum collections unlocks unprecedented opportunities for a diverse range of biological studies. Numerous studies proved the value of hDNA for a range of study cases, of vertebrate (Castañeda‐Rico et al. 2020; Huynh et al. 2023; Lyra et al. 2020) and invertebrate taxa (Derkarabetian et al. 2019; Gauthier et al. 2023; Habel et al. 2014; Mayer et al. 2021; Patzold et al. 2020). Our investigation indicated that a high initial amount of isolated DNA is beneficial in the context of historical DNA data recovery in insects. It was shown before that lower initial DNA concentration in isolates led to lower numbers of mapped reads, lower mean coverage, and smaller estimated library sizes, with greater adapter content in sequenced reads (McGaughran 2020). In case of museomics utilizing short‐read next generation sequencing, it thus seems recommendable to apply DNA isolation protocols that were designed to retain short DNA fragments (< 70 bp). It is to note, however, that high DNA amount alone is not necessarily predictive for library complexity, duplicate rates and locus recovery in all cases, like, for example, in ancient DNA studies where overall target abundance played a more important role (Enk et al. 2013). Future studies might in addition investigate whether the extraction of DNA from larger body parts yields more DNA and ultimately results in better sequence quality. However, because external structures such as legs or tarsi naturally desiccate more rapidly than larger internal tissues, DNA might be preserved more effectively in them and therefore remain advantageous for obtaining longer reads. However, the destruction of these often highly taxonomy‐informative morphological structures should be avoided. Additionally, different taxa of preserved insects might vary in the strength of sclerotization or exhibit diverging chemical baselines, which is why results might still differ between different beetle lineages. A more detailed understanding of the impact of these factors will allow to make the most out of the vast source of molecular information that is lying dormant in our natural history collections and help to improve best practice protocols for collectors and collection infrastructure.

## Author Contributions

**Daniel Lukic:** conceptualization (equal), data curation (equal), formal analysis (equal), investigation (equal), methodology (equal), visualization (lead), writing – original draft (equal). **Malte Petersen:** data curation (equal), formal analysis (equal), writing – review and editing (equal). **Jeel Patel:** data curation (equal), formal analysis (supporting), writing – review and editing (equal). **Rama S. Krovi:** formal analysis (equal), writing – review and editing (equal). **Dirk Ahrens:** conceptualization (equal), methodology (equal), resources (equal), writing – review and editing (equal). **Roland Gerstmeier:** conceptualization (equal), methodology (equal), resources (equal), writing – review and editing (equal). **Jonas Eberle:** conceptualization (equal), data curation (equal), formal analysis (equal), funding acquisition (lead), investigation (equal), methodology (equal), project administration (equal), resources (equal), supervision (equal), writing – review and editing (equal).

## Funding

This work was supported by the Austrian Science Fund (10.13039/501100002428), P36167.

## Conflicts of Interest

The authors declare no conflicts of interest.

## Supporting information


**Table S1:** Summary of beetle samples used in this study, including specimen metadata (ID, preservation type, country of origin, year and collector), taxonomic identification (subfamily, genus), NCBI accession numbers of raw reads, DNA quantity and quality metrics (amount of DNA in ng, most abundant DNA fragment length (the modal value of the fragment‐length distribution)), sequencing status and success (Seq. = sent for sequencing, Succ. = successfully sequenced), and sequencing outcomes (percentage of recovered loci of a total of 2897 loci, mean number of recovered base pairs, and mean gappiness).
**Table S2:** Genome size estimates of 10 studied specimens including specimen metadata (Sample‐ID, preservation method, year of collection and amount of gDNA gained from extraction) and taxonomic identification (genus, species).
**Table S3:** Comparison of ethanol‐ and dry‐preserved samples from individuals aged 5–16 years, a range in which samples were available for both preservation groups. Recovered loci are shown as the percentage of a total of 2897 reference loci. Mean loci length refers to the average length of recovered sequences excluding gaps. Gappiness is the percentage of gaps across all reference loci, averaged within each preservation group. Fragment length corresponds to the most abundant DNA fragment length (the modal value of the fragment‐length distribution). Pres. = preservation method.

## Data Availability

Metadata of employed specimens is provided are Supporting Information S1. DNA data are available from the Zenodo repository https://doi.org/10.5281/zenodo.17989238. The raw DNA and RNA sequencing data are available in the NCBI Sequence Read Archive (SRA) under BioProject accession number PRJNA1473665 (BioSample Accessions SAMN60575158–SAMN60575287). By improving the usability of historical collections, our work supports biodiversity research and conservation efforts globally. All methods and findings are shared openly to facilitate replication and application by other researchers and institutions.

## References

[ece374137-bib-0001] Allendorf, F. W. , W. C. Funk , S. N. Aitken , M. Byrne , G. Luikart , and A. Antunes . 2022. Conservation and the Genomics of Populations. Oxford University Press. 10.1093/oso/9780198856566.001.0001.

[ece374137-bib-0002] Andersen, J. C. , and N. J. Mills . 2012. “DNA Extraction From Museum Specimens of Parasitic Hymenoptera.” PLoS One 7, no. 10: e45549. 10.1371/journal.pone.0045549. 23077493 PMC3471897

[ece374137-bib-0003] Ávila‐Arcos, M. C. , E. Cappellini , J. A. Romero‐Navarro , et al. 2011. “Application and Comparison of Large‐Scale Solution‐Based DNA Capture‐Enrichment Methods on Ancient DNA.” Scientific Reports 1, no. 1: 74. 10.1038/srep00074. 22355593 PMC3216561

[ece374137-bib-0004] Bagley, J. C. , F. Alda , M. F. Breitman , E. Bermingham , E. P. van den Berghe , and J. B. Johnson . 2015. “Assessing Species Boundaries Using Multilocus Species Delimitation in a Morphologically Conserved Group of Neotropical Freshwater Fishes, the *Poecilia sphenops* Species Complex (Poeciliidae).” PLoS One 10, no. 4: e0121139. 10.1371/journal.pone.0121139. 25849959 PMC4388586

[ece374137-bib-0005] Bernstein, J. M. , and S. Ruane . 2022. “Maximizing Molecular Data From Low‐Quality Fluid‐Preserved Specimens in Natural History Collections.” Frontiers in Ecology and Evolution 10: 893088. 10.3389/fevo.2022.893088.

[ece374137-bib-0006] Booth, R. , and L. M. Crowley . 2025. “The Genome Sequence of a Chequered Beetle, *Tillus elongatus* (Linnaeus, 1758) (Coleoptera: Cleridae).” Wellcome Open Research 10: 575. 10.12688/wellcomeopenres.24678.1. 41287799 PMC12640499

[ece374137-bib-0007] Britz, R. , A. Hundsdörfer , and U. Fritz . 2020. “Funding, Training, Permits—The Three Big Challenges of Taxonomy.” Megataxa 1, no. 1: 1. 10.11646/megataxa.1.1.10.

[ece374137-bib-0086] Brown, M. E. , S. Ottati , and V. Trivellone . 2025. “A Non‐Destructive, Fast, Inexpensive, Non‐Toxic Chelating Resin‐Based DNA Extraction Protocol for Insect Voucher Specimens and Associated Microbiomes.” Journal of Insect Science 25, no. 3. 10.1093/jisesa/ieaf062. PMC12132034 40459989

[ece374137-bib-0008] Castañeda‐Rico, S. , L. León‐Paniagua , C. W. Edwards , and J. E. Maldonado . 2020. “Ancient DNA From Museum Specimens and Next Generation Sequencing Help Resolve the Controversial Evolutionary History of the Critically Endangered Puebla Deer Mouse.” Frontiers in Ecology and Evolution 8, no. 94: 94. 10.3389/fevo.2020.00094.

[ece374137-bib-0009] Chan, Y. L. , C. N. K. Anderson , and E. A. Hadly . 2006. “Bayesian Estimation of the Timing and Severity of a Population Bottleneck From Ancient DNA.” PLoS Genetics 2, no. 4: e59. 10.1371/journal.pgen.0020059. 16636697 PMC1440876

[ece374137-bib-0010] Chen, S. 2025. “Fastp 1.0: An Ultra‐Fast All‐Round Tool for FASTQ Data Quality Control and Preprocessing.” iMeta 4, no. 5: e70078. 10.1002/imt2.70078. 41112039 PMC12527978

[ece374137-bib-0011] Darriba, D. , M. Weiß , and A. Stamatakis . 2016. “Prediction of Missing Sequences and Branch Lengths in Phylogenomic Data.” Bioinformatics 32, no. 9: 1331–1337. 10.1093/bioinformatics/btv768. 26733454

[ece374137-bib-0012] Davis, C. C. , and S. Knapp . 2025. “Exploring Biodiversity Through Museomics.” Nature Reviews Genetics 26, no. 3: 149–150. 10.1038/s41576-024-00801-2. 39506143

[ece374137-bib-0013] Dean, M. D. , and J. W. O. Ballard . 2001. “Factors Affecting Mitochondrial DNA Quality From Museum Preserved *Drosophila simulans* .” Entomologia Experimentalis et Applicata 98, no. 3: 279–283. 10.1046/j.1570-7458.2001.00784.x.

[ece374137-bib-0014] Derkarabetian, S. , L. R. Benavides , and G. Giribet . 2019. “Sequence Capture Phylogenomics of Historical Ethanol‐Preserved Museum Specimens: Unlocking the Rest of the Vault.” Molecular Ecology Resources 19, no. 6: 1531–1544. 10.1111/1755-0998.13072. 31448547

[ece374137-bib-0015] Dessauer, H. C. , C. J. Cole , and M. S. Hafner . 1990. “Collection and Storage of Tissues.” In Molecular Systematics, edited by D. M. Hillis and C. Moritz , 29–47. Sinauer.

[ece374137-bib-0016] Dietz, L. , S. Kukowka , J. Eberle , et al. 2024. “Museomics Reveal Origins of East African *Pleophylla* Forest Chafers and Miocene Forest Connectivity.” Molecular Phylogenetics and Evolution 201: 108210. 10.1016/j.ympev.2024.108210. 39366592

[ece374137-bib-0017] Djernæs, M. , A. Bras , T. J. Simonsen , et al. 2026. “Optimization of DNA Extraction for Insect Museomics Substantially Increases DNA Yield.” European Journal of Entomology 123, no. 1: 48–60. 10.14411/eje.2026.008.

[ece374137-bib-0018] Enk, J. , J.‐M. Rouillard , and H. Poinar . 2013. “Quantitative PCR as a Predictor of Aligned Ancient DNA Read Counts Following Targeted Enrichment.” BioTechniques 55, no. 6: 301–309. 10.2144/000114114. 24344679

[ece374137-bib-0090] Fernández‐Ruiz, N. , S. Pinecki‐Socias , A. Estrada‐Peña , et al. 2023. “Decontamination Protocols Affect the Internal Microbiota of Ticks.” Parasites & Vectors 16, no. 1. 10.1186/s13071-023-05812-2. PMC10249182 37286996

[ece374137-bib-0019] Ferrari, G. , L. Esselens , M. L. Hart , et al. 2023. “Developing the Protocol Infrastructure for DNA Sequencing Natural History Collections.” Biodiversity Data Journal 11: e102317. 10.3897/BDJ.11.e102317. 38327316 PMC10848826

[ece374137-bib-0020] Fowler, E. V. , M. L. Starkie , M. J. Blacket , D. G. Mayer , and M. K. Schutze . 2024. “Effect of Temperature and Humidity on Insect DNA Integrity Evaluated by Real‐Time PCR.” Journal of Economic Entomology 117, no. 5: 1995–2002. 10.1093/jee/toae193. 39212660 PMC11473036

[ece374137-bib-0021] Gauthier, J. , M. Borer , E. F. A. Toussaint , J. Bilat , H. Kippenberg , and N. Alvarez . 2023. “Museomics Reveals Evolutionary History of *Oreina* Alpine Leaf Beetles (Coleoptera: Chrysomelidae).” Systematic Entomology 48, no. 4: 658–671. 10.1111/syen.12601.

[ece374137-bib-0022] Gilbert, M. T. P. , W. Moore , L. Melchior , and M. Worobey . 2007. “DNA Extraction From Dry Museum Beetles Without Conferring External Morphological Damage.” PLoS One 2, no. 3: e272. 10.1371/journal.pone.0000272. 17342206 PMC1803022

[ece374137-bib-0023] Gimenez, I. B. , C. J. Conti , and R. L. Cabrini . 1977. “The Effect of Decalcification on the Microspectrophotometric Determination of DNA.” Stain Technology 52, no. 6: 352–353. 10.3109/10520297709116813. 414380

[ece374137-bib-0024] Grabherr, M. G. , B. J. Haas , M. Yassour , et al. 2011. “Full‐Length Transcriptome Assembly From RNA‐Seq Data Without a Reference Genome.” Nature Biotechnology 29, no. 7: 7. 10.1038/nbt.1883. PMC3571712 21572440

[ece374137-bib-0087] Greenstone, M. H. , D. C. Weber , T. A. Coudron , M. E. Payton , and J. S. Hu . 2012. “Removing External DNA Contamination from Arthropod Predators Destined for Molecular Gut‐Content Analysis.” Molecular Ecology Resources 12, no. 3: 464–469. 10.1111/j.1755-0998.2012.03112.x. 22268594

[ece374137-bib-0025] Grewe, F. , M. R. Kronforst , N. E. Pierce , and C. S. Moreau . 2021. “Museum Genomics Reveals the Xerces Blue Butterfly ( *Glaucopsyche xerces* ) Was a Distinct Species Driven to Extinction.” Biology Letters 17, no. 7: 20210123. 10.1098/rsbl.2021.0123. 34283930 PMC8292013

[ece374137-bib-0026] Gu, X. 2024. Statistical Analysis of Molecular and Genomic Evolution. Oxford University Press. 10.1093/oso/9780198816515.001.0001.

[ece374137-bib-0027] Habel, J. C. , M. Husemann , A. Finger , P. D. Danley , and F. E. Zachos . 2014. “The Relevance of Time Series in Molecular Ecology and Conservation Biology.” Biological Reviews 89, no. 2: 484–492. 10.1111/brv.12068. 24251767

[ece374137-bib-0028] Harrison, L. , E. Kooienga , C. Speights , et al. 2020. “Microbial Succession From a Subsequent Secondary Death Event Following Mass Mortality.” BMC Microbiology 20, no. 1: 309. 10.1186/s12866-020-01969-3. 33050884 PMC7557037

[ece374137-bib-0029] Hawkins, M. T. R. , M. F. C. Flores , M. McGowen , and A. Hinckley . 2022. “A Comparative Analysis of Extraction Protocol Performance on Degraded Mammalian Museum Specimens.” Frontiers in Ecology and Evolution 10: 984056. 10.3389/fevo.2022.984056.

[ece374137-bib-0030] Hecht, B. C. , A. P. Matala , J. E. Hess , and S. R. Narum . 2015. “Environmental Adaptation in Chinook Salmon ( *Oncorhynchus tshawytscha* ) Throughout Their North American Range.” Molecular Ecology 24, no. 22: 5573–5595. 10.1111/mec.13409. 26465117

[ece374137-bib-0031] Heintzman, P. D. , S. A. Elias , K. Moore , K. Paszkiewicz , and I. Barnes . 2014. “Characterizing DNA Preservation in Degraded Specimens of *Amara alpina* (Carabidae: Coleoptera).” Molecular Ecology Resources 14, no. 3: 606–615. 10.1111/1755-0998.12205. 24266987

[ece374137-bib-0032] Holmquist, A. , H. Tavris , G. Kim , L. Esposito , B. Fisher , and A. Lam . 2025. “Towards Large‐Scale Museomics Projects: A Cost‐Effective and High‐Throughput Extraction Method for Obtaining Historical DNA From Museum Insect Specimens.” Molecular Ecology Resources 25, no. 8: e14117. 10.1111/1755-0998.14117. 40321019

[ece374137-bib-0088] Huszarik, M. , N. Röder , L. Eberhardt , et al. 2023. “External DNA Contamination and Efficiency of Bleach Decontamination for Arthropod Diet Analysis.” Environmental DNA 5, no. 3: 540–550. 10.1002/edn3.410.

[ece374137-bib-0033] Hutter, C. R. , and W. Duellman . 2023. “Filtration of Gene Trees From 9,000 Exons, Introns, and UCEs Disentangles Conflicting Phylogenomic Relationships in Tree Frogs (Hylidae).” Genome Biology and Evolution 15, no. 5: evad070. 10.1093/gbe/evad070. 37129064 PMC10171231

[ece374137-bib-0034] Huynh, S. , A. Cloutier , and S. Y. W. Sin . 2023. “Museomics and Phylogenomics of Lovebirds (Psittaciformes, Psittaculidae, *Agapornis*) Using Low‐Coverage Whole‐Genome Sequencing.” Molecular Phylogenetics and Evolution 185: 107822. 10.1016/j.ympev.2023.107822. 37220800

[ece374137-bib-0035] Johnson, K. R. , I. F. P. Owens , and The Global Collection Group . 2023. “A Global Approach for Natural History Museum Collections.” Science 379, no. 6638: 1192–1194. 10.1126/science.adf6434. 36952410

[ece374137-bib-0036] Kim, D. , L. Song , F. P. Breitwieser , and S. L. Salzberg . 2016. “Centrifuge: Rapid and Sensitive Classification of Metagenomic Sequences.” Genome Research 26, no. 12: 1721–1729. 10.1101/gr.210641.116. 27852649 PMC5131823

[ece374137-bib-0037] Kim, S. , and B. D. Farrell . 2025. “Target Enrichment Museomics of the Asian Long‐Horned Beetle and Its Relatives (Cerambycidae: *Anoplophora*) Reveals Two Independent Origins of Life in the Cold.” Systematic Entomology 50, no. 1: 139–153. 10.1111/syen.12647.

[ece374137-bib-0038] Klemm, P. , P. F. Stadler , and M. Lechner . 2023. “Proteinortho6: Pseudo‐Reciprocal Best Alignment Heuristic for Graph‐Based Detection of (Co‐)orthologs.” Frontiers in Bioinformatics 3: 1322477. 10.3389/fbinf.2023.1322477. 38152702 PMC10751348

[ece374137-bib-0039] Koski, L. B. , and G. B. Golding . 2001. “The Closest BLAST Hit Is Often Not the Nearest Neighbor.” Journal of Molecular Evolution 52, no. 6: 540–542. 10.1007/s002390010184. 11443357

[ece374137-bib-0040] Lechner, M. , S. Findeiß , L. Steiner , M. Marz , P. F. Stadler , and S. J. Prohaska . 2011. “Proteinortho: Detection of (Co‐)orthologs in Large‐Scale Analysis.” BMC Bioinformatics 12, no. 1: 124. 10.1186/1471-2105-12-124. 21526987 PMC3114741

[ece374137-bib-0041] Lim, G. S. , M. Balke , and R. Meier . 2012. “Determining Species Boundaries in a World Full of Rarity: Singletons, Species Delimitation Methods.” Systematic Biology 61, no. 1: 165–169. 10.1093/sysbio/syr030. 21482553

[ece374137-bib-0042] Lindahl, T. 1993. “Instability and Decay of the Primary Structure of DNA.” Nature 362, no. 6422: 709–715. 10.1038/362709a0. 8469282

[ece374137-bib-0089] Linville, J. , and J. Wells . 2002. “Surface Sterilization of a Maggot Using Bleach Does Not Interfere With Mitochondrial DNA Analysis of Crop Contents.” Journal of Forensic Sciences 47, no. 5: 1–5. 10.1520/jfs15532j. 12353545

[ece374137-bib-0043] Lopes, F. , N. Gunter , C. P. D. T. Gillett , et al. 2024. “From Museum Drawer to Tree: Historical DNA Phylogenomics Clarifies the Systematics of Rare Dung Beetles (Coleoptera: Scarabaeinae) From Museum Collections.” PLoS One 19, no. 12: e0309596. 10.1371/journal.pone.0309596. 39739893 PMC11687894

[ece374137-bib-0044] Lüdecke, D. , M. S. Ben‐Shachar , I. Patil , and D. Makowski . 2020. “Extracting, Computing and Exploring the Parameters of Statistical Models Using R.” Journal of Open Source Software 5, no. 53: 2445. 10.21105/joss.02445.

[ece374137-bib-0045] Lüdecke, D. , M. S. Ben‐Shachar , I. Patil , P. Waggoner , and D. Makowski . 2021. “Performance: An R Package for Assessment, Comparison and Testing of Statistical Models.” Journal of Open Source Software 6, no. 60: 3139. 10.21105/joss.03139.

[ece374137-bib-0046] Lyra, M. L. , A. C. C. Lourenço , P. D. P. Pinheiro , et al. 2020. “High‐Throughput DNA Sequencing of Museum Specimens Sheds Light on the Long‐Missing Species of the *Bokermannohyla claresignata* Group (Anura: Hylidae: Cophomantini).” Zoological Journal of the Linnean Society 190, no. 4: 1235–1255. 10.1093/zoolinnean/zlaa033.

[ece374137-bib-0047] MacDonald, H. 2025. “Conceptual Uncertainties and Practical Challenges in Voluntary Nagoya Protocol Compliance: The Australian Situation.” Biopreservation and Biobanking 23, no. 1: 23–30. 10.1089/bio.2024.0090. 39761123

[ece374137-bib-0048] Major, T. , P. Renk , J. Reissig , et al. 2023. “Museum DNA Reveals a New, Potentially Extinct Species of Rinkhals (Serpentes: Elapidae: Hemachatus) From the Eastern Highlands of Zimbabwe.” PLoS One 18, no. 9: e0291432. 10.1371/journal.pone.0291432. 37756254 PMC10529548

[ece374137-bib-0049] Mandrioli, M. , F. Borsatti , and L. Mola . 2006. “Factors Affecting DNA Preservation From Museum‐Collected Lepidopteran Specimens.” Entomologia Experimentalis et Applicata 120, no. 3: 239–244. 10.1111/j.1570-7458.2006.00451.x.

[ece374137-bib-0050] Marcelino, V. R. , E. C. Holmes , and T. C. Sorrell . 2020. “The Use of Taxon‐Specific Reference Databases Compromises Metagenomic Classification.” BMC Genomics 21, no. 1: 184. 10.1186/s12864-020-6592-2. 32106809 PMC7045516

[ece374137-bib-0051] Marinček, P. , N. D. Wagner , and S. Tomasello . 2022. “Ancient DNA Extraction Methods for Herbarium Specimens: When Is It Worth the Effort?” Applications in Plant Sciences 10, no. 3: e11477. 10.1002/aps3.11477. 35774991 PMC9215277

[ece374137-bib-0052] Martí, J. M. , C. R. Kok , J. B. Thissen , et al. 2024. “Addressing the Dynamic Nature of Reference Data: A New nt Database for Robust Metagenomic Classification.” *bioRxiv*. 10.1101/2024.06.12.598617. PMC12013259 40111052

[ece374137-bib-0053] Mayer, C. , L. Dietz , E. Call , S. Kukowka , S. Martin , and M. Espeland . 2021. “Adding Leaves to the Lepidoptera Tree: Capturing Hundreds of Nuclear Genes From Old Museum Specimens.” Systematic Entomology 46, no. 3: 649–671. 10.1111/syen.12481.

[ece374137-bib-0054] McGaughran, A. 2020. “Effects of Sample Age on Data Quality From Targeted Sequencing of Museum Specimens: What Are We Capturing in Time?” BMC Genomics 21, no. 1: 188. 10.1186/s12864-020-6594-0. 32111157 PMC7048091

[ece374137-bib-0055] Mende, M. B. , and A. K. Hundsdoerfer . 2013. “Mitochondrial Lineage Sorting in Action – Historical Biogeography of the *Hyles euphorbiae* Complex (Sphingidae, Lepidoptera) in Italy.” BMC Evolutionary Biology 13, no. 83: 83. 10.1186/1471-2148-13-83. 23594258 PMC3655913

[ece374137-bib-0056] Miller, W. , D. I. Drautz , J. E. Janecka , et al. 2009. “The Mitochondrial Genome Sequence of the Tasmanian Tiger ( *Thylacinus cynocephalus* ).” Genome Research 19, no. 2: 213–220. 10.1101/gr.082628.108. 19139089 PMC2652203

[ece374137-bib-0057] Mitchell, D. , E. Willerslev , and A. Hansen . 2005. “Damage and Repair of Ancient DNA.” Mutation Research 571, no. 1–2: 265–276. 10.1016/j.mrfmmm.2004.06.060. 15748652

[ece374137-bib-0058] Mullin, V. E. , W. Stephen , A. N. Arce , et al. 2023. “First Large‐Scale Quantification Study of DNA Preservation in Insects From Natural History Collections Using Genome‐Wide Sequencing.” Methods in Ecology and Evolution 14, no. 2: 360–371. 10.1111/2041-210X.13945.

[ece374137-bib-0059] Nunes, R. , C. Storer , T. Doleck , A. Y. Kawahara , N. E. Pierce , and D. J. Lohman . 2022. “Predictors of Sequence Capture in a Large‐Scale Anchored Phylogenomics Project.” Frontiers in Ecology and Evolution 10: 943361. 10.3389/fevo.2022.943361.

[ece374137-bib-0060] Ortiz, E. M. , A. Höwener , G. Shigita , et al. 2024. “A Novel Phylogenomics Pipeline Reveals Complex Pattern of Reticulate Evolution in Cucurbitales.” *bioRxiv* 2023.10.27.564367. 10.1101/2023.10.27.564367.

[ece374137-bib-0061] Pacheco, C. , D. Lobo , P. Silva , et al. 2022. “Assessing the Performance of Historical Skins and Bones for Museomics Using Wolf Specimens as a Case Study.” Frontiers in Ecology and Evolution 10: 970249. 10.3389/fevo.2022.970249.

[ece374137-bib-0062] Patzold, F. , A. Zilli , and A. K. Hundsdoerfer . 2020. “Advantages of an Easy‐To‐Use DNA Extraction Method for Minimal‐Destructive Analysis of Collection Specimens.” PLoS One 15, no. 7: e0235222. 10.1371/journal.pone.0235222. 32639972 PMC7343169

[ece374137-bib-0063] Pauli, M. T. , J. Gauthier , M. Labédan , M. Blanc , J. Bilat , and E. F. A. Toussaint . 2024. “Museomics of *Carabus* Giant Ground Beetles Shows an Oligocene Origin and In Situ Alpine Diversification.” Peer Community Journal 4: e70. 10.24072/pcjournal.445.

[ece374137-bib-0064] Pavlek, M. , J. Gauthier , V. Tonzo , J. Bilat , M. A. Arnedo , and N. Alvarez . 2022. “Life‐History Traits Drive Spatial Genetic Structuring in Dinaric Cave Spiders.” Frontiers in Ecology and Evolution 10: 910084. 10.3389/fevo.2022.910084.

[ece374137-bib-0065] Prosser, S. W. J. , J. R. deWaard , S. E. Miller , and P. D. N. Hebert . 2016. “DNA Barcodes From Century‐Old Type Specimens Using Next‐Generation Sequencing.” Molecular Ecology Resources 16, no. 2: 487–497. 10.1111/1755-0998.12474. 26426290

[ece374137-bib-0066] R Core Team . 2023. “R: A Language and Environment for Statistical Computing (Version 4.3.0) [Computer Software].” R Foundation for Statistical Computing. https://www.R‐project.org.

[ece374137-bib-0067] Rajora, O. P. (Ed.). 2019. Population Genomics: Concepts, Approaches and Applications. Springer International Publishing. 10.1007/978-3-030-04589-0.

[ece374137-bib-0068] Ranallo‐Benavidez, T. R. , K. S. Jaron , and M. C. Schatz . 2020. “GenomeScope 2.0 and Smudgeplot for Reference‐Free Profiling of Polyploid Genomes.” Nature Communications 11, no. 1: 1432. 10.1038/s41467-020-14998-3. PMC7080791 32188846

[ece374137-bib-0069] Raxworthy, C. J. , and B. T. Smith . 2021. “Mining Museums for Historical DNA: Advances and Challenges in Museomics.” Trends in Ecology & Evolution 36, no. 11: 1049–1060. 10.1016/j.tree.2021.07.009. 34456066

[ece374137-bib-0071] Ross, H. A. , S. Murugan , and W. L. Sibon Li . 2008. “Testing the Reliability of Genetic Methods of Species Identification via Simulation.” Systematic Biology 57, no. 2: 216–230. 10.1080/10635150802032990. 18398767

[ece374137-bib-0072] Roycroft, E. , C. Moritz , K. C. Rowe , et al. 2022. “Sequence Capture From Historical Museum Specimens: Maximizing Value for Population and Phylogenomic Studies.” Frontiers in Ecology and Evolution 10: 931644. 10.3389/fevo.2022.931644.

[ece374137-bib-0073] Ruppert, L.‐S. , G. Segelbacher , M. Staab , and N. Winiger . 2023. “Gauging DNA Degradation Among Common Insect Trap Preservatives.” Entomologia Experimentalis et Applicata 171, no. 3: 218–226. 10.1111/eea.13266.

[ece374137-bib-0074] Sayyari, E. , J. B. Whitfield , and S. Mirarab . 2017. “Fragmentary Gene Sequences Negatively Impact Gene Tree and Species Tree Reconstruction.” Molecular Biology and Evolution 34, no. 12: 3279–3291. 10.1093/molbev/msx261. 29029241

[ece374137-bib-0075] Seutin, G. , B. N. White , and P. T. Boag . 1991. “Preservation of Avian Blood and Tissue Samples for DNA Analyses.” Canadian Journal of Zoology 69, no. 1: 82–90. 10.1139/z91-013.

[ece374137-bib-0076] Simmons, M. P. 2012. “Misleading Results of Likelihood‐Based Phylogenetic Analyses in the Presence of Missing Data.” Cladistics 28, no. 2: 208–222. 10.1111/j.1096-0031.2011.00375.x. 34872185

[ece374137-bib-0077] Stanke, M. , M. Diekhans , R. Baertsch , and D. Haussler . 2008. “Using Native and Syntenically Mapped cDNA Alignments to Improve de Novo Gene Finding.” Bioinformatics 24, no. 5: 637–644. 10.1093/bioinformatics/btn013. 18218656

[ece374137-bib-0078] Stathopoulou, P. M. , E. Asimakis , and G. Tsiamis . 2021. “Enterobacter: One Bacterium—Multiple Functions.” In Area‐Wide Integrated Pest Management. CRC Press.

[ece374137-bib-0079] Stočes, D. , T. Wijacki , A. Knoll , T. Kopecký , and J. Šipoš . 2025. “Evaluating DNA Quality in Coleoptera and Lepidoptera: Impact of Fixation and Preservation in Various Trapping Methods.” Entomologia Experimentalis et Applicata 173, no. 8: 903–917. 10.1111/eea.13591.

[ece374137-bib-0080] Thomsen, P. F. , S. Elias , M. T. P. Gilbert , et al. 2009. “Non‐Destructive Sampling of Ancient Insect DNA.” PLoS One 4, no. 4: e5048. 10.1371/journal.pone.0005048. 19337382 PMC2660418

[ece374137-bib-0081] Tin, M. M.‐Y. , E. P. Economo , and A. S. Mikheyev . 2014. “Sequencing Degraded DNA From Non‐Destructively Sampled Museum Specimens for RAD‐Tagging and Low‐Coverage Shotgun Phylogenetics.” PLoS One 9, no. 5: e96793. 10.1371/journal.pone.0096793. 24828244 PMC4020769

[ece374137-bib-0082] Toussaint, E. F. A. , J. Gauthier , J. Bilat , et al. 2021. “HyRAD‐X Exome Capture Museomics Unravels Giant Ground Beetle Evolution.” Genome Biology and Evolution 13, no. 7: evab112. 10.1093/gbe/evab112. 33988685 PMC8480185

[ece374137-bib-0083] Vurture, G. W. , F. J. Sedlazeck , M. Nattestad , et al. 2017. “GenomeScope: Fast Reference‐Free Genome Profiling From Short Reads.” Bioinformatics 33, no. 14: 2202–2204. 10.1093/bioinformatics/btx153. 28369201 PMC5870704

[ece374137-bib-0084] Watanabe, M. E. 2015. “The Nagoya Protocol on Access and Benefit Sharing: International Treaty Poses Challenges for Biological Collections.” Bioscience 65, no. 6: 543–550. 10.1093/biosci/biv056.

[ece374137-bib-0085] Zimmermann, J. , M. Hajibabaei , D. C. Blackburn , et al. 2008. “DNA Damage in Preserved Specimens and Tissue Samples: A Molecular Assessment.” Frontiers in Zoology 5: 18. 10.1186/1742-9994-5-18. 18947416 PMC2579423

