# Supplementary material for: Exploring the Quality of Historic DNA From Pinned Beetles
Source: Ecol Evol. 2026 Aug 14;16(8):e74137. doi: 10.1002/ece3.74137 (PMC13473914; doi:10.1002/ece3.74137)
Supplement: Supplementary file 1 [file ECE3-16-e74137-s001.pdf]

Supplement Table S1. Summary of beetle samples used in this study, including specimen metadata (ID, preservation type, country of origin, year and collector), taxonomic identification (subfamily, genus), NCBI accession numbers of raw reads, DNA quantity and quality metrics (amount of DNA in ng, most abundant DNA fragment length (the modal value of the fragment-length distribution)), sequencing status and success (Seq. = sent for sequencing, Succ. = successfully sequenced), and sequencing outcomes (percentage of recovered loci of a total of 2,897 loci, mean number of recovered base pairs, and mean gappiness).

ID	Pres.	Country	Year	Collector	Subfamily	Genus	accession	DNA (ng)	Frag. length (bp)	Seq.	Succ.	percent of rec. loci	Mean loci length (bp)	Gappiness
JE73	dry	Italy	1988	Werner	Clerinae	*Trichodes*		84.864						
JE74	dry	Russia	1989	R. Cervenka	Clerinae	*Trichodes*		5.184						
JE78	dry	Turkey	2000	B. Baume	Clerinae	*Trichodes*		8.256						
JE79	dry	Turkey	2000	B. Baume	Clerinae	*Trichodes*	SAMN60575158	11.232		x	x			
JE80	dry	Austria	1987	J. Wolf	Clerinae	*Trichodes*		93.408						
JE81	ethanol	China	2008	Weigel				31.392						
JE82	ethanol	Greece	2011	Eberle & Gerstmeier	Clerinae	*Trichodes*	SAMN60575159	717.12		x	x			
JE83	ethanol	Greece	2011	Eberle & Gerstmeier	Clerinae	*Trichodes*	SAMN60575160	406.08		x	x			
JE84	dry	Jordan	1994	Becvar	Clerinae	*Trichodes*		36.096						
JE85	dry	Iran	1999	L. Bieber	Clerinae	*Trichodes*		146.88						
JE86	ethanol	Israel	2023	Eberle/Lukic	Clerinae	*Trichodes*	SAMN60575161	453.12		x	x			
JE87	ethanol	Israel	2023	Eberle/Lukic	Clerinae	*Trichodes*		1027.2						
JE88	dry	Greece - Macedonia	1980	K. & S. Wellschmied	Clerinae	*Trichodes*		13.92						
JE89	dry	Turkey	1977	K. & S. Wellschmied	Clerinae	*Trichodes*		16.608						
JE90	dry	Israel	1981	Roland Gerstmeier	Clerinae	*Trichodes*	SAMN60575162	390.72		x	x			
JE91	dry	Turkey	1990	H. Kocağağ	Clerinae	*Trichodes*	SAMN60575163	1564.8		x	x			
JE92	dry	North Macedonia	1989	Meiser	Clerinae	*Trichodes*	SAMN60575164	14.496		x	x			
JE93	dry	Bulgaria	1982	R. Bláha	Clerinae	*Trichodes*	SAMN60575165	24.288		x	x			
JE94	ethanol	Indonesia	2009		Clerinae	*Omadius*	SAMN60575166	563.52		x	x			

JE95	ethanol	Venezuela	2009	Wachtel	Clerinae	*Priocera*	SAMN60575167	338.88		x	x			
JE104	dry	Turkey	1984	Wellschmied	Clerinae	*Trichodes*	SAMN60575168	21.024	110	x	x	5.73	344.49	0.61
JE106	dry	Turkey	1993	V. Bíža & Z. Košťál	Clerinae	*Trichodes*	SAMN60575169	378.24	78	x	x	84.26	611.68	0.49
JE107	dry	S. Turkey	1984	R. Frieser	Clerinae	*Trichodes*	SAMN60575170	118.08	69	x	x	43.11	357.08	0.66
JE108	dry	Kazakhstan	2001	M. Hauser	Clerinae	*Trichodes*	SAMN60575171	51.744	71	x	x	7.28	275.56	0.75
JE109	dry	Greece	1990	Heiss	Clerinae	*Trichodes*	SAMN60575172	31.296	72	x	x	5.90	273.86	0.71
JE113	dry	Tajikistan	1989		Clerinae	*Trichodes*	SAMN60575173	30.336	59	x	x	2.45	302.15	0.71
JE115	dry	Uzbekistan	1989	Wrzecionko	Clerinae	*Trichodes*	SAMN60575174	48.768	67	x	x	13.60	349.29	0.70
JE117	dry	Spain	1975	Mager & Mühle	Clerinae	*Trichodes*	SAMN60575175	37.632	74	x	x	4.38	233.98	0.77
JE118	dry	Tajikistan	1989	Wrzecionko	Clerinae	*Trichodes*	SAMN60575176	600.96	72	x	x	77.29	484.37	0.56
JE119	dry	USA	1988	Eric van den Berghe	Clerinae	*Trichodes*	SAMN60575177	139.2	69	x	x	17.26	347.53	0.69
JE120	dry	USA	1983	Hobbard	Clerinae	*Trichodes*	SAMN60575178	443.52	74	x	x	45.60	366.76	0.66
JE121	dry	Iran	2010	J. Weipert	Clerinae	*Trichodes*	SAMN60575179	134.4	110	x	x	29.55	326.28	0.68
JE122	dry	Turkey	1997	Ma. Halada	Clerinae	*Trichodes*	SAMN60575180	340.8	68	x	x	7.11	288.64	0.71
JE123	dry	Turkey	1983	Roland Gerstmeier	Clerinae	*Trichodes*	SAMN60575181	340.8	52	x	x	0.28	220.13	0.75
JE124	dry	China	2001	Jaroslav Turna	Clerinae	*Trichodes*	SAMN60575182	327.36	71	x	x	0.07	130.50	0.88
JE125	dry	Turkey	1978	de Freina	Clerinae	*Trichodes*	SAMN60575183	1641.6	67	x	x	95.03	833.93	0.34
JE126	dry	Tanzania	2013	local collector	Clerinae	*Trichodes*	SAMN60575184	253.44	72	x	x	74.73	518.58	0.55
JE127	dry	Ethiopia	2002	Werner & Sudre	Clerinae	*Trichodes*	SAMN60575185	58.848	63	x	x	25.13	326.53	0.68
JE128	dry	Turkey	1996	Brechtel & Ehrmann TÜ-Arl	Clerinae	*Trichodes*	SAMN60575186	126.72	59	x	x	9.29	315.89	0.67
JE129	dry	Spain	1984	Schacht	Clerinae	*Trichodes*	SAMN60575187	50.112		x	x	94.17	824.39	0.35
JE130	dry	Turkey	2001	Hiermeier	Clerinae	*Trichodes*	SAMN60575188	17.664	61	x	x	10.25	301.66	0.71
JE131	dry	Turkey	1985	W. Schacht	Clerinae	*Trichodes*	SAMN60575189	1593.6		x	x	97.62	972.68	0.26
JE132	dry	Spain	1988	W. Schacht	Clerinae	*Trichodes*	SAMN60575190	155.52	75	x	x	97.83	999.01	0.24
JE133	ethanol	Indonesia	2009		Clerinae	*Omadius*	SAMN60575191	1152	8661	x	x	79.70	865.68	0.34
JE134	ethanol	Indonesia	2009		Clerinae	*Omadius*	SAMN60575192	1440	7537	x	x	77.63	821.92	0.37
JE135	ethanol	Indonesia	2009		Clerinae	*Stigmatium*	SAMN60575193	1142.4	8661	x	x	81.71	911.21	0.32

JE136	ethanol	Indonesia	2009		Clerinae	*Wilsonoclerus*	SAMN60575194	1622.4	6805	x	x	81.12	886.44	0.34
JE137	ethanol	Indonesia	2009		Clerinae	*Stigmatium*	SAMN60575195	1353.6	7965	x	x	82.36	910.52	0.32
JE138	ethanol	Indonesia	2009		Clerinae	*Stigmatium*	SAMN60575196	1132.8		x	x	81.88	904.75	0.32
JE139	ethanol	Indonesia	2009		Clerinae	*Stigmatium*	SAMN60575197	419.52		x	x	81.77	908.79	0.32
JE140	ethanol	Indonesia	2009		Clerinae	*Stigmatium*	SAMN60575198	544.32		x	x	82.19	909.56	0.32
JE141	ethanol	China	2008	Weigel	Clerinae	*Clerus*	SAMN60575199	11.616		x	x	9.87	269.49	0.75
JE142	ethanol	China	2009	Weigel	Clerinae		SAMN60575200	404.16		x	x	83.50	83.50	924.59
JE143	ethanol	China	2009	Weigel	Hydnocerinae	*Callimerus*	SAMN60575201	1036.8		x	x	82.60	893.64	0.32
JE144	ethanol	China	2009	Weigel	Orthopleurinae	*Tenerus*	SAMN60575202	265.92		x	x	81.26	920.10	0.31
JE145	ethanol	Germany	2010	Tilo	Korynetinae	*Korynetes*	SAMN60575203	1507.2		x	x	84.92	907.45	0.31
JE146	ethanol	Iran	2011	Mühle	Korynetinae	*Necrobia*	SAMN60575204	368.64		x	x	84.43	911.65	0.31
JE147	ethanol	Austria	2012	A. Gruppe	Clerinae	*Opilo*	SAMN60575205	2851.2		x	x	90.65	986.73	0.26
JE148	ethanol	Italy	2012	R. Gerstmeier	Dasytinae	*Henicopus*	SAMN60575206			x	x			
JE149	ethanol	Mongolia	2012	A. Gruppe	Clerinae	*Trichodes*	SAMN60575207	3110.4		x	x	98.03	1063.58	0.20
JE150	ethanol	Indonesia	2010			*Callimerus*	SAMN60575208	1046.4		x	x	83.53	914.47	0.32
JE151	ethanol	Indonesia	2010		Clerinae	*Wilsonoclerus*	SAMN60575209	1545.6		x	x	75.39	948.15	0.30
JE152	ethanol	Indonesia	2010			*Thalerocnemis*	SAMN60575210	906.24		x	x	81.39	905.62	0.32
JE153	ethanol	Indonesia	2010		Clerinae	*Wilsonoclerus*	SAMN60575211	1228.8		x	x	80.05	883.06	0.33
JE154	ethanol	Indonesia	2010			*Omadius*	SAMN60575212	998.4		x	x	81.91	894.91	0.33
JE155	ethanol	Indonesia	2010			*Callimerus*	SAMN60575213	514.56		x	x	76.39	823.98	0.36
JE156	ethanol	Indonesia	2010		*Tarsosteninae*			SAMN60575214		x	x	82.36	82.36	922.20
JE157	ethanol	Greece	2011		Clerinae	*Trichodes*	SAMN60575215	1987.2	6864	x	x	98.34	1050.71	0.21
JE158	ethanol	Greece	2009	A. Gruppe		*Thanasimus*	SAMN60575216	3580.8		x	x	89.44	986.73	0.27
JE160	ethanol	Malaysia peninsula	2023	A. Skale		*Cladiscus*	SAMN60575217	387.84		x	x	81.91	910.91	0.32
JE161	ethanol	Malaysia peninsula	2023	A. Skale	Orthopleurinae	*Tenerus*	SAMN60575218	1017.6		x	x	79.88	915.45	0.31
JE162	ethanol	Malaysia peninsula	2023	A. Skale	Tillinae	*Cylidrus*	SAMN60575219	715.2		x	x	78.98	892.89	0.33
JE163	ethanol	Germany	2023	P. Seifert		*Necrobia*	SAMN60575220	2294.4		x	x	84.81	944.95	0.30

JE164	ethanol			R. Gerstmeier			SAMN60575221	7.104		x	x	1.38	226.65	1.38
JE165	ethanol			R. Gerstmeier		*Gyponyx*		SAMN60575222		x	x	85.09	85.09	906.02
JE166	ethanol		2020	Ziegler G.		*Necrobia*	SAMN60575223	248.64		x	x	79.46	844.54	0.34
JE167	ethanol	Germany	2023	Ziegler G.	Tillinae	*Tillus*	SAMN60575224	3868.8		x	x	81.81	935.67	0.31
JE168	ethanol	Greece	2009	A. Gruppe		*Thanasimus*	SAMN60575225	3340.8		x	x	89.40	985.62	0.26
JE171	dry	S Yemen	2001			*Wittmeridecus*	SAMN60575226	170.88		x	x	78.49	851.83	0.35
JE172	dry	S Namibia	2010	W. Schawaller	Tillinae	*Eucymatodera*	SAMN60575227	233.28		x	x	78.22	871.11	0.34
JE173	dry	Namibia	2016	R. Gerstmeier	Tillinae	*Eucymatodera*	SAMN60575228	565.44		x	x	43.94	364.91	0.65
JE174	dry	Kenya	1999	M. Snizek	Tillinae	*Eucymatodera*	SAMN60575229	478.08		x	x	76.01	782.40	0.38
JE175	dry	Morocco	2000	H. Mühle	Clerinae	*Trichodes*	SAMN60575230	370.56	97	x	x	71.73	460.24	0.60
JE176	dry	Morocco mer.	1995	M. Snizek	Clerinae	*Trichodes*	SAMN60575231	108.48	64	x	x	1.28	282.81	0.72
JE177	dry	Cyprus	1993	Egger	Clerinae	*Trichodes*	SAMN60575232	44.256	67	x	x	1.31	278.84	0.73
JE178	dry	The Republic of South Africa	1999	M. Halada	Clerinae	*Trichodes*	SAMN60575233	527.04	88	x	x	76.63	521.01	0.55
JE179	dry	Turkey	2000	M. Halada	Clerinae	*Trichodes*	SAMN60575234	1545.6	76	x	x	75.77	495.98	0.57
JE182	dry	Vietnam	1986	J. Rybnicek	Clerinae	*Trichodes*	SAMN60575235	666.24	62	x	x	22.68	339.67	0.68
JE183	ethanol				Tillinae		SAMN60575236	2323.2		x	x	78.39	78.39	823.07
JE184	ethanol					*Graptoclerus*	SAMN60575237	1372.8		x	x	74.53	672.20	0.46
JE186	dry	Armenia	1989	Z. Kejval	Clerinae	*Trichodes*	SAMN60575238	73.632	60	x	x	17.71	368.01	0.67
JE187	dry	South Africa	2000	U. Schmidel	Clerinae	*Trichodes*	SAMN60575239	353.28	83	x	x	81.36	556.43	0.52
JE190	dry	Mexico	2009	Eric M. Fischer	Clerinae	*Trichodes*	SAMN60575240	3225.6	725	x	x	97.96	1051.78	0.21
JE191	dry	Iran	2004	Mühle	Clerinae	*Trichodes*	SAMN60575241	901.44	63	x	x	34.21	336.58	0.68
JE192	dry	Austria	2023	Walter Wallner	Clerinae	*Clerus*	SAMN60575242	1248		x	x	46.91	366.23	0.65
JE195	dry	Ethiopia	2005		Clerinae	*Trichodes*	SAMN60575243	2361.6	63	x	x	69.90	460.90	0.59
JE196	dry			Martin Lahadj	Clerinae	*Trichodes*	SAMN60575244	89.568		x	x	1.62	292.79	0.72
JE197	dry			Attica Reitter	Clerinae	*Trichodes*	SAMN60575245	1411.2		x	x	45.67	394.40	0.65

JE198	dry		1984	St. Zimmermann	Clerinae	*Trichodes*	SAMN60575246	464.64		x				
JE199	dry	USA	1979	T. Griswold	Clerinae	*Trichodes*	SAMN60575247	1468.8	63	x	x	67.41	422.88	0.62
JE200	dry	USA	1965	D.C. Arnold	Clerinae	*Trichodes*	SAMN60575248	434.88	45	x	x	0.72	255.86	0.75
JE201	dry	Kenya	1991	Werner	Clerinae	*Trichodes*	SAMN60575249	868.8	69	x	x	66.41	428.84	0.61
JE202	dry	Iran	2008	Mühle	Clerinae	*Trichodes*	SAMN60575250	1497.6	67	x	x	97.72	979.86	0.25
JE203	dry			Erinsan Korb	Clerinae	*Trichodes*	SAMN60575251	2563.2		x	x	0.52	308.80	0.69
JE204	dry	Kazakhstan		F. Hauser	Clerinae	*Trichodes*		SAMN60575252		x	x	27.89	27.89	351.10
JE205	dry	Armenia	2004	Kalashian	Clerinae	*Trichodes*	SAMN60575253	259.2	68	x	x	8.53	292.53	0.75
JE207	dry	Morocco	1985	Lesne	Clerinae	*Trichodes*	SAMN60575254	590.4	57	x	x	14.98	304.62	0.72
JE208	dry	USA	1963	G. H. Nelson	Clerinae	*Trichodes*	SAMN60575255	505.92	56	x	x	53.50	365.92	0.65
JE209	dry	Spain	1964	M. Ortego	Clerinae	*Trichodes*	SAMN60575256	615.36	55	x	x	0.07	112.50	0.89
JE210	dry	Iran	1999	V. Major	Clerinae	*Trichodes*	SAMN60575257	763.2	63	x	x	27.30	387.74	0.66
JE211	dry	Uzbekistan	1976	Kr. Pospíšil	Clerinae	*Trichodes*	SAMN60575258	45.408	54	x	x	0.45	286.62	0.75
JE212	dry	Kazakhstan		St. Zimmermann	Clerinae	*Trichodes*		SAMN60575259		x	x	80.19	80.19	544.95
JE213	dry			St. Zimmermann	Clerinae	*Trichodes*	SAMN60575260	1958.4		x	x	15.57	352.36	0.68
JE214	dry	Algeria		A. Otto	Clerinae	*Trichodes*	SAMN60575261	1305.6		x	x	1.45	306.64	0.69
JE215	dry	Iran	1968	St. Zimmermann	Clerinae	*Trichodes*	SAMN60575262	253.44	74	x	x	23.61	348.39	0.68
JE216	dry	Pakistan	1982	Eckweiler	Clerinae	*Trichodes*	SAMN60575263	960	55	x	x	60.86	411.33	0.62
JE217	dry	Turkey	1968	K. Kusdas	Clerinae	*Trichodes*	SAMN60575264	851.52	65	x	x	74.63	473.55	0.58
JE218	dry	Afghanistan	1952	J. Klapperich	Clerinae	*Trichodes*	SAMN60575265	413.76	59	x	x	36.04	369.67	0.66
JE219	dry			St. Zimmermann	Clerinae	*Trichodes*	SAMN60575266	3043.2		x	x	66.00	434.10	0.61
JE220	dry	Tunisia		B. v. Bodemeyer	Clerinae	*Trichodes*	SAMN60575267	696		x	x	24.75	390.86	0.65
JE221	dry	USA	1945	Horn	Clerinae	*Trichodes*	SAMN60575268	1958.4	47	x	x	8.25	282.45	0.74
JE222	dry	Transkaspia (Kazakhstan?)	1907	Hauser	Clerinae	*Trichodes*	SAMN60575269	1008	52	x	x	28.96	364.22	0.66
JE223	dry	Afghanistan	1971	Kabakov	Clerinae	*Trichodes*	SAMN60575270	179.52	60	x	x	64.72	432.82	0.61

JE224	dry	Iran	2018	Murastyi D.	Clerinae	*Trichodes*	SAMN60575271	312.96	98	x	x	96.03	833.93	0.34
JE225	ethanol	Greece	2011	Eberle & Gerstmeier	Clerinae	*Trichodes*	SAMN60575272	1977.6	7000	x	x	97.96	1045.73	0.21
JE226	ethanol	Greece	2011	Eberle & Gerstmeier	Clerinae	*Trichodes*	SAMN60575273	2150.4	10226	x	x	97.83	1036.11	0.22
JE228	dry	North Macedonia	1988	I. Wolf	Clerinae	*Trichodes*	SAMN60575274	1891.2	54	x	x	96.17	845.38	0.33
JE229	ethanol	Israel	2023	Eberle/Lukic	Clerinae	*Trichodes*	SAMN60575275	1382.4	10089	x	x	97.79	1018.47	0.23
JE230	ethanol				Clerinae	*Thanasimus*	SAMN60575276	259.2		x	x	80.01	845.72	0.35
JE231	dry	Iran	2017	Jaroslav Dalihod	Clerinae	*Trichodes*	SAMN60575277	174.72	71	x	x	24.99	355.65	0.66
JE235	ethanol	Germany	2012	R. Trusch	Clerinae	*Trichodes*	SAMN60575278	496.32		x	x	98.14	1009.73	0.24

Supplement Table S2. Genome size estimates of 10 studied specimens including specimen metadata (Sample-ID, preservation method, year of collection and amount of gDNA gained from extraction) and taxonomic identification (genus, species).

Sample	Genome haploid length min (bp)	Genome haploid length max (bp)	Genus	Species	Pres.	Coll. year	DNA (ng)
JE79	313.62	412.05	*Trichodes*	sp.	dry	2000	11.232
JE82	1385.39	1474.61	*Trichodes*	*favarius*	EtOH	2011	717.12
JE83	556.09	574.70	*Trichodes*	*punctatus*	EtOH	2011	406.08
JE86	1302.87	1336.87	*Trichodes*	*affinis*	EtOH	2023	453.12
JE90	108.15	111.69	*Trichodes*	sp.	dry	1981	390.72
JE91	442.65	462.35	*Trichodes*	sp.	dry	1990	1564.8
JE92	309.74	1004.16	*Trichodes*	*crabroniformis*	dry	1989	14.496
JE93	0.47	NA	*Trichodes*	*favarius*	dry	1982	24.288
JE94	719.50	737.91	*Omadius*	*omissus*	EtOH	2009	563.52
JE95	925.30	978.37	*Priocera*	sp.	EtOH	2009	338.88

Supplementary Table S3. Comparison of ethanol- and dry-preserved samples from individuals aged 5–16 years, a range in which samples were available for both preservation groups. Recovered loci are shown as the percentage of a total of 2,897 reference loci. Mean loci length refers to the average length of recovered sequences excluding gaps. Gappiness is the percentage of gaps across all reference loci, averaged within each preservation group. Fragment length corresponds to the most abundant DNA fragment length (the modal value of the fragment-length distribution). Pres. = preservation method.

Pres.	No. samples	Recovered loci (%)	Mean loci length (bp)	Gappiness (%)	Sample age (a)	Fragment length (bp)	DNA (ng)
ethanol	28	85.2	933	29.9	14.1	7970	1404
dry	7	63.6	617	48.9	10.7	215	715
